# Internet, unmet aspirations and the U-shape of life

**DOI:** 10.1371/journal.pone.0233099

**Published:** 2020-06-05

**Authors:** Fulvio Castellacci, Henrik Schwabe

**Affiliations:** University of Oslo, Oslo, Norway; University of Glasgow, UNITED KINGDOM

## Abstract

The relationship between age and well-being is U-shaped. One recent explanation for this empirical pattern is related to unmet aspirations theory, pointing out that optimism bias decreases life satisfaction at younger ages, whereas pessimism bias increases it at later stages of life. This paper investigates the effects of Internet use on subjective well-being over the life cycle. Our model investigates the proposition that Internet use affects aspirations, and that this effect is relatively stronger at younger and older ages. To investigate moderation effects of Internet use on the U-shape of life, we use the Eurobarometer annual surveys for the years 2010 to 2016, which provide rich information for around 150,000 individuals in all European countries. We focus on the *EU Digital Agenda* policy program, and exploit exogenous variation in broadband Internet take-up across European countries to identify the causal effects of Internet on life satisfaction for different age groups. The results of 2SLS estimations for a recursive bivariate ordered probit model show that active Internet users have a different well-being pattern over the life cycle compared to less active users. Specifically, we find that Internet use makes the U-shape of life steeper. Country-level evidence on aspiration levels for different demographic and Internet user groups indicates that our empirical results are consistent with unmet aspirations theory.

## 1. Introduction

The literature on subjective well-being has pointed out a variety of factors that explain differences in happiness conditions reported by individuals [1–7]. A new strand of research has recently extended this literature and started to investigate what effects Internet may have on individuals’ well-being. The introduction of the Internet variable in the study of subjective well-being is warranted. Digital technologies are pervasive, and nowadays most individuals use the Internet to access information and communicate with each other. It is reasonable to think that online information and communication patterns may affect individuals’ aspirations and their perceived well-being [8].

A small number of studies have recently presented first empirical investigations of this question. Some of these works have analyzed the overall relationship between Internet adoption and life satisfaction using survey data for several countries, and pointed out an overall positive correlation between the two variables for large samples of individuals [9–11]. Other papers have made use of country-specific surveys to investigate more specific hypotheses, such as Internet effects related to material aspirations and social comparisons [12, 13], and those related to the use of social media and communication patterns [14–16].

Overall, the main endeavor of this recent strand of research has been to uncover a general relationship between Internet use and well-being for large samples of individuals, but there has until now been more limited effort to investigate the extent to which this relationship varies for different groups of individuals. Specifically, it is reasonable to postulate that individuals of different ages use the Internet to a varying extent and for distinct purposes. The effects of online digital technologies on well-being may indeed vary substantially with age.

This is the point that we seek to investigate in the present paper. The work presents an empirical analysis of the relationships between Internet use intensity and subjective well-being, and its specific objective is to study how this relationship varies over the life cycle. The investigation of this question is closely related to extant research on the U-shaped relationship between age and well-being [17, 18]. According to this literature, subjective well-being over the life cycle is characterized by a remarkable empirical regularity, with a progressive decline in life satisfaction until late adult life, followed by a steady recovery and growth in subsequent years.

Recent research has put forward a new explanation of the U-shape relationship based on “unmet aspirations theory” [19]. This argues that individuals make systematic forecast errors when they form expectations about future life satisfaction, and that these errors indicate optimism bias at early stages of life, and pessimism bias at older ages [20–22]. Since unmet aspirations depress life satisfaction for younger individuals, and, by contrast, unanticipated well-being fosters life satisfaction for older people, this theory can explain the U-shape pattern that has been observed and confirmed in previous empirical studies.

We take this theory as a conceptual framework for our analysis, and we extend it by investigating the effects of Internet use. We present a simple model, extending Schwandt [19], in which Internet use increases individuals’ aspirations, and relatively more so for more vulnerable age groups, such as younger and older individuals [23]. Our model predicts that Internet use makes the U-shape relationship between age and well-being steeper, i.e. exacerbating optimism bias for the younger and pessimism bias for the older.

To empirically investigate this hypothesis, we make use of the Eurobarometer survey, a large survey that covers several thousand individuals in all European countries. We use the six annual surveys that refer to the years 2010 to 2016, which provide a rich dataset for about 150,000 individuals. Our identification strategy is based on the *EU Digital Agenda* policy program, which was initiated in the late 2000s to foster digitalization and Internet access in member countries [24]. The EU Digital Agenda provides an exogenous source of variation in broadband Internet take-up across European countries and regions. In fact, as we will show in the paper, the implementation of the EU policy program in different countries and regions in Europe during the period 2010–2016 was characterized by substantial spatial and temporal variation, and the timing of the roll-out was not related to other factors driving life satisfaction and its correlates.

We exploit this exogenous variation to identify the causal effects of Internet on life satisfaction for different age groups. Our instrumental variables–lagged fixed broadband take-up–measures *peer effects* in Internet adoption, based on the idea that the intensity of Internet use of each individual will partly depend on the overall level of diffusion of broadband Internet in the country or region [11, 25, 26]. The econometric analysis estimates a 2SLS recursive bivariate ordered probit model, which simultaneously estimates a treatment and an outcome equation using the CMP procedure developed by Roodman [27].

The main result of this analysis is that the effects of Internet use on subjective well-being are heterogeneous, varying significantly with age. The econometric findings point out that active Internet users have a more pronounced decrease in reported life satisfaction in their younger adult life, an earlier and stronger recovery after the turning point of the U-shape, and steady growth throughout older adult life. In short, we find that Internet use makes the U-shape of life steeper. We then provide empirical evidence that illustrates that this empirical result is consistent with the predictions of unmet aspirations theory, and the model presented in this paper.

On the whole, the contribution of this paper to the literature is twofold. First, we contribute to the recent strand of research on Internet and well-being by showing that the effects of Internet are remarkably heterogeneous among individuals of different ages. This means that age-specific characteristics must be taken into account when analyzing benefits and risks that the Internet leads to. Second, we contribute to the literature on the U-shaped relationship between age and well-being by introducing a new conceptual dimension, the Internet, and by formally testing its moderation effects on the age-well-being relationship.

## 2. Literature

A new strand of research has recently begun to investigate the effects of Internet use on life satisfaction and subjective well-being, carrying out econometric analyses of large survey datasets (see overview in Castellacci and Tveito [8]). Two groups of studies contribute to this emerging literature. In the first group, papers typically make use of cross-country surveys (e.g. European Social Survey, Eurobarometer, Gallup World Poll) to provide estimates of the average correlation between Internet use and life satisfaction for a large sample of individuals in different countries.

Kavetsos and Koutrompis [28] analyze the relationships between mobile phones, computers with Internet connection and life satisfaction using the Eurobarometer dataset for all European countries for the years 2005–2008. OLS estimates point out a positive correlation between computers with Internet connection and life satisfaction. Penard et al. [11], using data from the European Value Survey for Luxembourg in 2008, study the effects of Internet use on life satisfaction. The 2SLS cross-sectional estimates reported in this paper do not find any significant effect when other relevant control variables are included in the regressions. Graham and Nikolova [10] carry out a cross-country study of the relationship between Internet access and life satisfaction, using data from the Gallup World Poll for a large number of world economies in the period 2009–2011. Ordered logit cross-sectional correlation coefficients reported in this paper are positive and significant, and they vary substantially across world regions.

The second group of studies comprises papers that analyze large national household surveys, which often provide more specific variables to measure different types of online activities and Internet-related use, and thus enable to test more elaborated hypotheses. As noted below, many of these studies point out negative (or moderating) effects that Internet use has on well-being through its interactions with income and relational factors.

Focusing first on the relational dimension, Rotondi et al. [15] study the effects of smartphone use on relational patterns and social life, employing data from the Italian Multipurpose Survey on Households. 2SLS estimates point out a positive effect of smartphone use on life satisfaction, but they also show that this effect is weaker for those individuals that use the smartphone in combination with face-to-face social activities. Sabatini and Sarracino [16] investigate the relationships between social media use, social capital and well-being, using the Italian Multipurpose Survey on Households. 2SLS and SEM results reported in this article point out a significant negative effect of social media use on subjective well-being.

Regarding income-related effects, Lohmann [12] analyzes the hypothesis that Internet has negative effects on well-being by raising individuals’ material aspirations. The work uses data from various sources, and specifically the German Socio-Economic Panel, the EU-SILC survey and the World Value Survey. OLS and ordered probit cross-sectional estimates reported in this paper indicate a positive and significant correlation between Internet use and life satisfaction, but also corroborate the hypothesis that Internet raises material aspirations and so weakens the positive effect of income on subjective well-being. Finally, Sabatini and Sarracino [13] investigate a similar mechanism using the Italian Multipurpose Survey on Households. The study finds in particular that use of social media spurs social comparisons and raises income aspirations, thus moderating income-related effects on subjective well-being.

In short, this recent strand of research points out a variety of different results regarding the impact of Internet use on subjective well-being, some emphasizing positive effects and others suggesting negative impacts. A common characteristic of this literature is that nearly all papers seek to uncover a general relationship between Internet use and well-being for the whole sample of individuals in the dataset, but they do not investigate the extent to which this relationship may vary for different groups of individuals. In other words, extant research has until now had limited interest in the study of the heterogeneity of effects of Internet.

In this paper, we seek to study heterogeneous effects of Internet with respect to age. A few previous studies on this topic provide mixed evidence. Research focusing on Internet use of younger adults (and particularly on the use of social media and video games) point out both positive and negative effects [8, 29, 30]. Kross et al. [23] present evidence from experience-sampling analysis of a small group of Facebook users in the US, showing the negative effects that this has on the users’ well-being. McDool et al. [14] study the effects of social media use and children’s well-being, using the Household Longitudinal Survey for the UK, and find negative effects for their sample of British children. Arad et al. [31] focuses on Facebook users in a security-related organization, and point out that social media increases social comparison and weakens subjective well-being for the younger half of their sample. Arampatzi et al. [32]) study a large sample of Dutch social media users, and show that the negative effects on well-being is particularly strong for SNS users who also report to be feel socially disconnected and lonely.

On the other hand, research focusing on Internet use of older adults is still scant, and it so far indicates that Internet use has mostly positive effects on well-being by facilitating social contacts and communication, and thus decreasing isolation and depression [33–36].

In order to provide a more systematic understanding of Internet effects for individuals of different age groups, we turn to the literature on the U-shaped relationship between age and well-being. Blanchflower and Oswald [17, 18] point out the existence of a U-shape relationship between age and life satisfaction, a remarkable regularity that holds for a large number of countries worldwide. According to these studies, the turning point of the U-shape–i.e. the year at which individuals face a so-called midlife crisis–is between 35 and 65 years (depending on countries, sample, and model specifications). Some studies argue that the U-shape is a methodological artefact that can be explained by cohort effects, and/or by the use of inappropriate control variables [37, 38], and Frijters and Beatton [39] point out that the U-shape may be affected by the omission of fixed effects in pooled cross-country regressions. In spite of these methodological remarks, the empirical evidence in support of the U-shaped relationship between age and life satisfaction is strong.

The literature has recently advanced a possible explanation of this empirical regularity. This is related to “unmet aspirations” [19]. A variety of empirical studies within psychology, neuroscience and behavioral economics has provided evidence that individuals make systematic forecast errors when they form expectations about the future, and that these prediction errors change along the life cycle. Optimism bias prevails at younger ages, whereas pessimism bias is more common at older life stages [20–22] (p. 123). According to this theory, the mismatch between expectations and realized life satisfaction may explain the observed decrease in well-being during the first part of life, and the corresponding increase at older ages. A relevant related study is Proto and Rustichini (2015), which show that life satisfaction depends on the gap between aspired and realized income, and that this relationship is moderated by individuals’ personality traits such as neuroticism.

## 3. Theoretical framework

The simple model presented in this section provides the theoretical framework for the empirical analysis. The model is based upon a generalization of Schwandt’s [19] recent study of life satisfaction and unmet aspirations, and it extends his framework to investigate the effects of Internet use. In line with the literature on the determinants of well-being, we assume that individuals derive life satisfaction from different *domains of life* (e.g. working life, consumption, social life). Individual *i*’s life satisfaction at time *t* is given by the following equation:

$${LS}_{t}=\sum_{d=1}^{D}d_{t}[x(d,t)]-\lambda_{t}•\sum_{\tau =0}^{t-t_{0}}\{E_{t-\tau -1}[{LS}_{t-\tau }]-{LS}_{t-\tau }\}$$ (1)

The first term on the RHS of the equation is the sum of life satisfaction derived from all domains of life *d* at time *t*. Each domain of life’s satisfaction is achieved by means of the vector of commodities (or life achievements) *x*(*d*,*t*). The function *d*_*t*_ can in principle change over the life cycle, because individuals can change their preferences over time and may therefore derive different life satisfaction levels from the same vector of commodities at different ages.

The second term represents a forecast error that individuals make in their expectation formation about future life satisfaction. According to Schwandt [19], individuals expect their future life satisfaction to be a function of their current satisfaction level. If this expectation is wrong, the resulting forecast error (unmet aspiration) will affect their current life satisfaction level. Specifically, when forecast errors are positive (negative), individuals systematically predict that their future life satisfaction will be higher (lower) than it will actually prove to be. In other words, at any given period *t*, an individual makes expectations about her future life satisfaction level at time *t+1*. Then, at time *t+1*, the individual will compare her expectations with the actual realized life satisfaction level. Unmet aspirations are given by the sum of all past (positive) forecast errors, starting from early life (t0–1; in the notation adopted in Eq 1), until the current period.

Such unmet aspirations will have a negative effect on the individual’s current level of well-being, because they will lead to frustration and regret about unrealized objectives and achievements that the individual had previously hoped to realize. In the second term of Eq 1, *λ*_*t*_ is a so-called *regret* parameter, representing the negative effect of forecast error on current life satisfaction. The parameter ranges between 0 and 1; the higher it is, the stronger is the regret for past failures (or unmet aspirations). For simplicity, we assume here that the regret parameter is fixed and it does not vary with age. Bertoni and Corazzini [21] interestingly point out that regret might actually be asymmetric, tending to be stronger when people make negative forecast errors (optimism bias) than when they make positive errors (pessimism bias). This refinement would however not change qualitatively the main properties and predictions of this model, which we discuss below.

The expectation that an individual forms at time *t-τ-1* about her life satisfaction at time *t-τ* is given by the expression:

$$E_{t-\tau -1}[{LS}_{t-\tau }]=[1+\beta_{t-\tau -1}]•{LS}_{t-\tau -1}$$ (2)

Future expectations are positively affected by the variable *β*_*t*_, which represents individuals’ *aspirations* about the future. In line with recent empirical evidence [19, 21, 22], we postulate that aspirations will decrease over the life cycle, and that they will be positive (*optimism bias*) at younger ages until midlife (*t* < *t(M*)), and become negative (*pessimism bias*) at later stages of life (beyond midlife; *t* > *t(M*)).

$$\frac{\partial \beta (t)}{\partial t}<0;\beta (t)=\{\begin{array}{l} \beta >0,\;t<t(M)\\ \beta <0,\;t>t(M) \end{array}$$ (3)

Introducing this expectation term in Eq 1 yields:

$${LS}_{t}=\sum_{d=1}^{D}d_{t}[x(d,t)]-\lambda_{t}•\sum_{\tau =0}^{t-t_{0}}\{[1+\beta_{t-\tau -1}]•{LS}_{t-\tau -1}-{LS}_{t-\tau }\}$$ (4)

Writing the extended form for all periods, and solving for *LS*_*t*_ gives the following expression:

$${LS}_{t}=\frac{\sum_{d=1}^{D}d_{t}[x(d,t)]}{1-\lambda_{t}}-\frac{\lambda_{t}}{1-\lambda_{t}}•\sum_{\tau =1}^{t-t_{0}}\beta_{t-\tau }•{LS}_{t-\tau }$$ (5)

The first term on the RHS of this equation is the sum of life satisfaction that an individual derives from the set of commodities and achievements that she has available from all domains of life *d*, divided by the complement to 1 of the regret parameter. This term is hence magnified by the regret parameter: the higher the regret for past failures, the more important current satisfaction with domains of life will become for an individual’s well-being (i.e. an individual with high regret must compensate these unmet aspirations with current life status). The second term on the RHS is the *optimism bias* term. As noted above, this term is affected by aspirations and it decreases over the life cycle.

This simple model can generate a U-shaped relationship between age and well-being in two ways. The first is related to the second term on the RHS of Eq 5, namely through optimism bias and unmet aspirations. As shown by Schwandt [19], if the aspiration variable *β* decreases over the life cycle–so that younger (older) people systematically overestimate (underestimate) their future life satisfaction–then the life satisfaction variable would follow a U-shape, because unmet aspirations would depress well-being at young ages, whereas the opposite mechanism would foster well-being later in life.

A second way in which this model could generate a U-shaped relationship arises if life satisfaction related to one given domain of life changes over the life cycle following a non-monotonic relationship. Specifically, assuming for simplicity that the forecast error term is 0, this happens if the function *d(t)* is U-shaped, i.e. because individuals derive a satisfaction level from domain of life *d* that decreases until midlife, and then rises again at older ages. For instance, this would be the case if individuals change some of their preferences over the life cycle, e.g. valuing differently the importance of their social life. This could be regarded as an important dimension of life satisfaction at younger ages, less important during midlife (when many individuals shift their focus to working life and career objectives), and then becoming again more important at later stages of life.

In both of the cases noted here (decreasing aspirations or changing preferences over the life cycle), the resulting relationship between life satisfaction and age would be U-shaped.

### Effects of Internet use on aspirations

To investigate the effects of Internet use in this framework, suppose now that we have two types of individuals, one that is a high (active) Internet user (HI), and the other that is low (sporadic) Internet user (LI). Based on extant literature, we argue that the different intensity of Internet use between these individuals may affect the U-shaped relationship between age and well-being. Active Internet use will expose individuals to a variety of online content that will foster social comparison mechanisms, and thereby affect individuals’ expectation formation and raise their aspiration levels [12, 13]. According to extant literature on the effects of Internet on well-being, such online comparison mechanisms and the related aspiration effects will be different for distinct age groups. Aspiration effects may in fact be relatively stronger for more vulnerable age groups.

In particular, in line with recent empirical evidence from various studies, we posit that younger individuals using the Internet are highly exposed to social comparisons and peer effects through the use of social media, which will tend to foster their material aspirations [13, 14, 23, 31, 32]. On the other hand, to the extent that older adults have more pessimistic expectations about the future, exposure to online content about bad news and threats (criminality, political and economic crises, terrorism, climate change) will end up exacerbating such pessimism bias [40, 41]. If this is the case, the aspiration variable *β*_*t*_ will have a steeper (negative) trend for the group of active Internet users than for sporadic users:

$$|{\beta_{t}}^{HI}|>\left|{\beta_{t}}^{LI}\right|$$ (6)

Further, it may also be argued that digital communication technologies will make the elderly more socially connected and less isolated than they had previously expected to be, and this may lead to a realized life satisfaction that exceeds the one that was previously expected [35, 42]. Under these conditions, it follows that Internet use will make the U-shape relationship between life satisfaction and age steeper. This means that the decrease in life satisfaction at early stages of life (before the turning point *t*(*M*)) would become more pronounced for active Internet users due to stronger optimism bias, and that, correspondingly, the increase in life satisfaction after midlife (*t* > *t*(*M*)) would also become swifter because of stronger pessimism bias:

### Proposition 1

$$\beta_{t}=\left\{ \begin{array}{c} \frac{{\partial LS(t)}^{HI}}{\partial t}<\frac{{\partial LS(t)}^{LI}}{\partial t},\;t<t(M)\\ \frac{{\partial LS(t)}^{HI}}{\partial t}>\frac{{\partial LS(t)}^{LI}}{\partial t},\;t>t(M) \end{array}\right.$$

This is the proposition that will be empirically tested and discussed in the subsequent sections.

## 4. Data and methods

The empirical analysis makes use of the Eurobarometer survey, a large survey that covers several thousand individuals in all European countries. We use the annual surveys that refer to the years 2010 to 2016, which provide a pooled cross-sectional dataset for around 150,000 individuals. These surveys provide harmonized data for the main variables of interest, so that we can analyze variables that have the exact same formulation in the Eurobarometer questionnaire. The Eurobarometer surveys are representative of the country population. They collect data by carrying out face-to-face interviews in people's home and in the national language”. Life satisfaction is measured by asking respondents to indicate their level of satisfaction on a four-point scale ranging from not very satisfied to very satisfied. The internet use variable is based on responses to the following question: “How often do you use the internet?”. Responses are given on a seven-point scale (no internet access: 7%; never: 21%; less often: 2%; two or three times a month: 1%; about once a week: 3%; two or three times a week: 9%; everyday/almost everyday: 57%). A1 Table presents a list of the variables we use in the empirical analysis, and some descriptive statistics for the whole dataset.

The econometric analysis seeks to investigate the effect of Internet use on life satisfaction for individuals of different age groups. An important issue that has to be taken into account is that the main explanatory variable of interest, Internet use intensity, is arguably not an exogenous and randomly assigned variable, but it is in turn dependent on a set of personal characteristics that define individuals’ willingness and capability to use the Internet. Some of these personal characteristics may be unobserved, and they may in principle affect both the treatment variable Internet use and the outcome variable life satisfaction.

To take this issue into account, we adopt a two-equation instrumental variable approach. The first step is a selection equation that investigates the factors explaining why some individuals have higher Internet use intensity than others, whereas the second equation studies the relationship between life satisfaction and Internet use (plus a set of control factors). The econometric model (baseline specification) is the following:

$$LS_{ict}=\alpha +\gamma INT_{ict}+\delta X_{ict}+\eta_{c}+\theta_{t}+\varepsilon_{ict}$$ (7)

$$INT_{ict}=\lambda +\rho X_{ict}+\mu Z_{ict}+\sigma_{ict}$$ (8)

where LS stands for life satisfaction, INT is internet use intensity, and **X** is a vector of covariates (control variables). The sub-indexes *i*, *c* and *t* indicate individuals, countries and years respectively. η_*c*_ is a set of country-specific effects, and θ_*t*_ is a set of time dummies. The variable Z in Eq (8) is an exogenous instrumental variable that is correlated with INT but not correlated with the error term of the outcome equation, as explained further below.

An identification strategy for this model has to consider two aspects. The first one is to formulate a comprehensive specification of Eq 8, and in particular include in the vector **X** all variables that are known to affect Internet use intensity according to extant literature on the subject. We thus include the following factors previously pointed out in studies of the determinants of Internet adoption and use: age, gender, occupation type, education level, civil status, and geographical location (urban vs. rural area), along with a set of country-specific effects and time dummies. This is a comprehensive set of variables that are supposedly able to account for a substantial part of the variability of Internet use intensity [43, 44]. The second aspect is to find an instrumental variable Z that provides exogenous variation correlated with the treatment variable Internet use intensity but uncorrelated with the error term of the outcome equation. For this purpose, we exploit cross-country differences in broadband take-up among European nations and regions as a source of exogenous variation.

In the late 2000s, the EU initiated a new policy to foster access and use of broadband Internet in European countries. The *EU Digital Agenda*, introduced in May 2010, formulated a set of objectives that national Member States have to achieve to increase their degree of digitalization, and particularly their Broadband Internet take-up [45]. However, *National Broadband Plans* that have subsequently been developed by Member States to implement the Digital Agenda have been quite different among European nations [24], and policy targets have been met at different speeds. Cross-country differences have been substantial on both the supply- and the demand-side. On the one hand, the supply of broadband infrastructures is affected by national regulation and competition policies that define entry costs and investment rates of telecommunication firms [46]. Supply-side factors are also related to geographical factors and pre-existing phone and Internet infrastructures, which affect the pace at which broadband transmission centrals and local access points are built up in different regions and countries. Recent papers have adopted an econometric identification strategy centered on supply-side factors, and in particular on the availability of broadband infrastructures, and its differences across municipalities and regions within a given country [16, 47–51] [15]. On the other hand, demand-side factors are also characterized by marked cross-country differences, linked for instance to different rates of diffusion of e- public services, as well as institutional differences in national education systems and public investments in e-skills [24].

**Fig 1:**
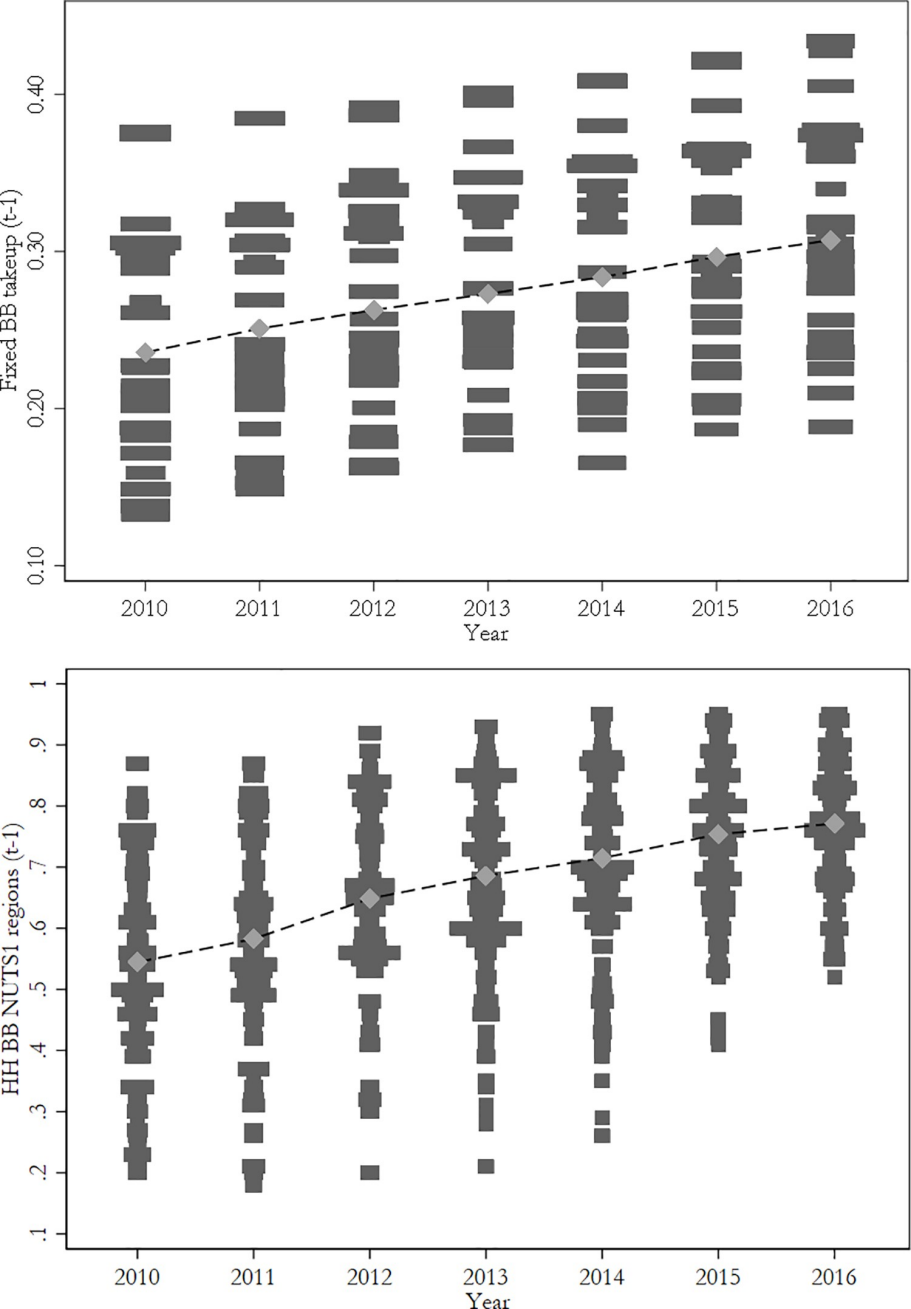
Distribution of fixed broadband across EU countries (above) and regions (below).

Fig 1 shows the distribution of broadband Internet take-up across countries and regions in Europe, and its evolution during the time span considered in this paper. The graphs show that spatial and temporal variation in broadband coverage is substantial. Using this source of variation to identify the causal effects of Internet use on life satisfaction relies upon two features of the EU digital policy program [48] (p. 1250). First, that the supply and demand factors that affected the development of broadband Internet take up during this period did not vary substantially over time; second, that the timing of the broadband Internet expansion does not co-vary with other factors that are correlated with the outcome variable life satisfaction. We will empirically assess these two assumptions in the next sub-section.

Based on this exogenous source of variation, we make use of two instrumental variables that measure the diffusion of broadband Internet (fixed broadband take-up per 100 people) in European countries and regions during the period: one is defined at the country-level, and the other is defined at the regional level. We take lagged values of these variables (one year before each survey period) in order to ensure that they predate the outcome variable and they are thus uncorrelated with common country- or region-year shocks [52] (p. 192–197). We carried out all estimations and robustness tests using the two instruments in separate exercises. The baseline results we present in the manuscript are those for the fixed broadband take-up at the country-level, and the additional results reported in the appendix are those for the corresponding variable measured at the regional level.

Note that our instrumental variables are “peer effects” [52], based on the idea that the intensity of Internet use of each individual will not only depend on the set of personal characteristics specified in equation 10, but also on the overall level of diffusion of broadband Internet in the country (or region). The idea that peer effects affect Internet use intensity is in line with standard models of diffusion of ICTs (see e.g.) [42]. These models argue that an individual is more likely to adopt and actively use new digital technologies if many other individuals have previously adopted and used the same technology. The reason is threefold: (1) *social learning*: adoption is easier if individuals can learn from other peers about the potential of the new technology; (2) *social pressure*: if most other peers are using a new digital technology (e.g. for communication purposes), it is hard for an individual not to use the same digital tool; (3) *network externalities*: since adoption and use costs depend on the size of users’ network, the larger the number of peers using a digital technology the cheaper this will be for a given user [11, 26, 53].

The key assumption of this identification strategy is that these instrumental variables affect individual life satisfaction only through their impact on Internet use intensity, and that they are therefore uncorrelated with any possible unobserved determinant of life satisfaction. This is a reasonable assumption, since for each individual *i* in our dataset, the extent of the diffusion of broadband Internet in the country, or region, in which *i* lives is determined by a set of dimensions that cannot be affected by each individual (and particularly so since our instruments *predate* the individual-level outcome variable). The next sub-sections will empirically assess the validity of this assumption for our dataset.

After clarifying the identification strategy, we now briefly point out the methods that we will use to estimate the effects of Internet use for individuals of different age groups, and in particular to test moderation effects of Internet on the U-shaped relationship between age and well-being. We expect the use of Internet may affect (1) the turning point of the U-shape (i.e. the time at which the midlife crisis sets in on average), and/or (2) the slope of the U-shaped curve (i.e. the speed at which the midlife crisis is met, and the subsequent recovery phase for older adults). We test these hypotheses by inserting two interaction terms in the regressions: one is an interaction between age and Internet use, and the other is an interaction between age-squared and Internet use. When we include such interaction variables in the regressions, we instrument them by using the corresponding interactions between the instrument and the age and age-squared variables, respectively. The interaction between age-squared and Internet use is the variable of our interest, using which we can test the two distinct moderation effects noted above (for further details on how to test moderation effects of U-shaped relationships, see 53: 1187).

We estimate Eqs (7) and (8) through a 2SLS recursive bivariate ordered probit model, which simultaneously estimates the two equations adopting an ordered probit approach, given the categorical nature of both the outcome and treatment variables [54, 55]. The recursive bivariate probit is a seemingly unrelated regression model with correlated disturbances, in which the dependent variable of the first equation appears on the right-hand-side of the second equation. The model is estimated by MLE. Greene [56] (p. 715–716) points out that in such a model the endogeneity of the RHS variable of the second equation could in principle be neglected because this term does not affect the maximization of the log-likelihood (differently from what it would be the case in a linear recursive model not estimated by MLE). We estimate this 2SLS recursive bivariate ordered probit model using the CMP procedure developed by Roodman [27] All our regressions are weighted, using a combined post-stratification and population size weight specified by the Eurobarometer for each respondent.

Since our instrument is measured at the national (or regional) level, the estimations are exposed to grouped structures [52] (p. 308–315). We therefore cluster our standard errors at the regional level when the instrumental variable is defined at this level of aggregation (see results in the appendix). On the other hand, when the instrumental variable is defined at the country level, it is more appropriate not to cluster standard errors due to the limited number of national groups in our data [57].

## 5. Results

### First stage results and LATE analysis

Before presenting the estimation results for the first stage, we seek to shed further light on the variability of our instrumental variables. First, we further assess the assumption that the timing of the broadband Internet expansion does not co-vary with other factors that are correlated with the outcome variable life satisfaction. A2 Table presents the results of regressions in which the dependent variable is the annual growth rate of the instrumental variable, and the RHS variables are the country average of the control variables at the beginning of the period. A2 Table shows that the timing of broadband Internet expansion is uncorrelated with the set of control variables that supposedly affect life satisfaction (e.g. education, occupation type, gender, age, marital status, financial situation, unemployment).

In A3 Table, we report the results of some balancing regressions (OLS), in which the dependent variables are the main socio-economic control variables in the model (income status, education, unemployment), and on the RHS we include future broadband take-up (in addition to lagged take-up and other control variables). The results indicate that the estimated coefficient for future take-up is not significant, ruling out further the possibility that broadband Internet expansion co-varies with other determinants of life satisfaction.

A4 Table presents the results of the first stage estimations (Eq 8), in which the dependent variable is the intensity of Internet use of each individual. The baseline results presented in the first column of A4 Table show that the control variables take the sign as expected based on previous literature on the determinants of adoption and use of the Internet [43, 44]. The table indicates that Internet use intensity decreases with age. It is higher for people in the workforce that have higher education level and white-collar occupations, and that report a good financial situation. Unemployed workers have lower Internet use intensity than average, arguably because they do not use the Internet for professional activities. Further, Internet use is on average higher for individuals that live in a large town as opposed to those in a rural area.

The most important finding in A4 Table is that the instrumental variable–lagged value of the country’s fixed broadband take-up–is as expected positively and significantly related to Internet use intensity. We obtain the same result when the instrumental variable is defined at the regional level (see A2 Table). As explained in the previous section, this finding corroborates the importance of “peer effects” as a determinant of Internet use, i.e. based on the idea that an individual is more likely to adopt and actively use Internet if many other individuals in the same country (or region) have previously adopted and used it, due to social learning, social pressure and/or network externality effects [11, 25, 26].

Our first stage estimates have a local average treatment effects (LATE) interpretation [52]. They represent the effect of fixed broadband take-up on the sub-population of *compliers* in each country, i.e. individuals that intensify their Internet use when a larger number of individuals in that country have actively been using Internet in the previous two years. Following the approach used in recent papers [48, 50], we carry out an analysis of the characteristics of the complier group. In A5 Table, we report the estimated coefficients of the effect of the instrumental variable on Internet use intensity (first stage regressions using a linear IV model) for different age sub-groups.

The table shows that individuals that respond more actively to increases in fixed broadband infrastructures are middle-aged adults (between 25 and 54), and less so younger and older Internet users. The pattern for middle-aged age groups is reasonable, since individuals in this stage of life typically use fixed broadband Internet as a professional tool in their working life, as well as for a variety of different uses related to their family and social life. On the other hand, the result that younger individuals (15–24 years old) increase their Internet use only marginally when their peers do is somewhat surprising. A possible explanation of this pattern is that younger people in our sample are those that in the period 2010–2016 increasingly begun to use wireless mobile broadband, which gradually substituted the use of fixed broadband. Hence, it may simply be the case that our instrument (based on fixed broadband development) underestimates compliers effects for adolescents and young Internet users because it neglects the early phase of diffusion of mobile broadband.

### Second stage results

A6 Table presents the results of the second stage (Eq 7), in which the dependent variable is the life satisfaction reported by each individual. We briefly discuss the results for the control variables first, before turning to the effect of the main variables of interest. The estimated results for the control variables are in line with extant research on the determinants of subjective well-being [1, 2, 4, 6]. A6 Table indicates that the relationship between age and life satisfaction is U-shaped, with lowest reported subjective well-being for middle-aged individuals. We will elaborate further on this U-shaped relationship later in this section. Among other control variables, highly educated individuals report higher life satisfaction than less educated people; unemployed individuals substantially lower satisfaction levels than employed people; and life satisfaction is higher for those individuals that have a good financial situation.

The top part of A6 Table reports the estimation results for the main variable of interest: Internet use intensity. This has a positive and significant effect on life satisfaction. Marginal effects of the Internet use variable, not reported here, are positive and significant too. The next columns in the table report some additional tests in order to assess the robustness of this result and the validity of our identification strategy. First, the second column of A6 Table includes an additional control variable–the country-level time trend of life satisfaction in the period before the broadband Internet expansion. The inclusion of this additional regressor does not change the result for the Internet use variable, which is still positive and significant. This rules out the possibility that the positive effect of Internet use on life satisfaction is driven by pre-existing trends in outcome (we have carried out the same test and obtained the same result for region-specific life satisfaction time trends).

Second, the third column reports the results of a placebo test, which tests whether Internet take-up at *t+1* affects current life satisfaction at time *t*. In this placebo regression, we exclude the Internet use variable and correspondingly include the lead value of the instrumental variable (i.e. measured one year *after* the life satisfaction and other control variables). The future broadband take-up variable is not significant in the estimations, ruling out the possibility that our results are driven by some omitted variables that are related to both life satisfaction and broadband Internet expansion.

Third, the fourth column of A6 Table includes an additional set of control variables, cohort effects, in order to test whether the U-shape relationship between age and well-being is a methodological artefact driven by the omission of cohort effects in life satisfaction regressions [38, 58]. The estimation results, though, are in line with the other findings in the Table: life satisfaction is U-shaped in age, and Internet has a direct positive effect on the dependent variable.

Finally, after presenting these robustness tests, we shift the focus to the point of our main interest: the moderation effects of Internet on the U-shaped relationship between age and well-being. We test these moderation effects by introducing two interaction terms in the regression model: (1) Internet * age; and (2) Internet * age-squared. The latter is the main interaction variable of our interest, providing a direct test of moderation effects of Internet use on the U-shape of life [53]. The fifth column of A6 Table reports the full model specification that includes such interaction terms. The estimated coefficient of the variable Internet * age-squared is positive and significant, confirming our hypothesis that Internet use moderates the U-shaped relationship between age and well-being. We also computed marginal effects of this interaction variable by comparing predicted probabilities for the two polar cases of Internet use (everyday vs. never) for different values of the age squared variable. In line with the estimated coefficient reported in A6 Table, the marginal effect of the interaction variable is positive and significant. The slope analysis reported below in this section will elaborate further on this result. A7 Table provides similar evidence by running the same 2SLS oprobit regressions separately for four sub-groups of individuals (15–24; 25–39; 40–54; 55+). The results confirm that the effects of Internet use on life satisfaction are positive and significant, but they vary substantially with age.

Moderation effects of Internet on the age-well-being relationship can have two forms: (1) Internet use can affect the location of the turning point of the U-shape–i.e. changing the time at which, on average, individuals begin to experience a recovery period after midlife crisis; and/or (2) Internet use can change the curvature of the U-shape, making it flatter or steeper–i.e. changing the speed at which individuals fall into midlife crisis and recover thereafter. From a conceptual point of view, the latter effect is more interesting and relevant than the former. However, the two effects are obviously related to each other, and we will therefore analyze them both.

A8, A9 and A10 Tables report the results of tests of these moderation effects. First, A8 Table presents the results of second-stage estimations for seven sub-samples, each defined by a distinct Internet use intensity (i.e. sub-sample 1 (sub-sample 7) only refers to those individuals that report no Internet use (highest Internet use)). The estimated coefficients for the age and age-squared variables in A8 Table indicate that the slope and curvature of the U-shape of life change as we move from lower to higher levels of Internet use intensity.

We calculated the turning points of the U-shape (i.e. age of midlife crisis) for the seven regressions reported in A8 Table. A9 Table reports the turning point for different levels of the Internet use intensity, and it shows that this moves towards the left as Internet use intensity increases (from around 53 to 50 years old), meaning that active Internet users, on average, begin a recovery period after the midlife crisis somewhat *earlier* than individuals who use Internet less actively.

A10 Table shifts the focus to the second type of moderation effect, which is the one of our main interest. The table reports the estimated slope of the U-shape at six different ages (25, 35, 45, 55, 65, 75 and 85) and for each Internet use intensity level (1 to 7). We also tested the slope difference for four different years around the turning point (following the method described in Haans et al. [53]: 1195). The slopes reported in A10 Table show a clear and consistent pattern. First, the slopes are as expected negative before the turning point and positive thereafter. Second, and more relevant, a comparison of the magnitudes of slopes between different Internet user groups shows that Internet use intensity makes the U-shaped relationship between age and life satisfaction *steeper*. This finding is also consistent with the positive sign of the estimated interaction effect Internet * age-squared previously reported in A6 Table. This means that Internet use accelerates the decline in life satisfaction that characterizes young adults and middle-aged individuals until the midlife crisis; and that it strengthens the subsequent growth and recovery period for older adults. After the turning point of the U-shape, active Internet users turn out to experience a much more pronounced and rapid recovery from the midlife crisis, reporting steadily increasing levels of life satisfaction.

## 6. Discussion

What can explain these empirical results? The simple theoretical framework outlined in section 3 points to the role of unmet aspirations [19, 22], and how these are affected by Internet use for different age groups. The main idea put forward in the model is twofold. First, the framework assumes that aspirations decrease over the life cycle, leading to optimism bias in the first part of life, and pessimism bias at older ages [21]. It is such decreasing trend of aspirations that explains a U-shaped relationship between age and well-being. Second, our model points out that if aspirations decline over time more rapidly for Internet users than non-users, Internet use would make the U-shape of life steeper (which is precisely the main empirical result that has been shown by the regression analysis above).

In our dataset, we are not able to empirically test these two properties of the model at the individual level (differently from Schwandt [19] who had available a panel dataset that enabled the test of this assumption). However, the Eurobarometer survey dataset has a few questions about individuals’ expectations on their future life satisfaction that can be used to provide aggregate evidence on the relevance of unmet aspirations theory. Specifically, we have used the following four variables that can be constructed based on survey questions about individuals’ expectations on how their life will be in the following 12 months: (1) life as a whole; (2) working life; (3) financial situation; (4) social life. Each variable is defined on a 1–3 scale (1: worse than today; 2: same as today; 3: better than today). Since our dataset is not a panel, we are not able to match individuals’ responses to the life satisfaction and expectations questions at time *t* (which would provide a direct measure of unmet aspirations at the individual level). Instead, we have collapsed these four expectation variables at the country-level, and calculated their average for different age groups and different Internet users groups, in order to provide aggregate evidence on the relationships between expectations, life satisfaction and Internet use.

**Fig 2:**
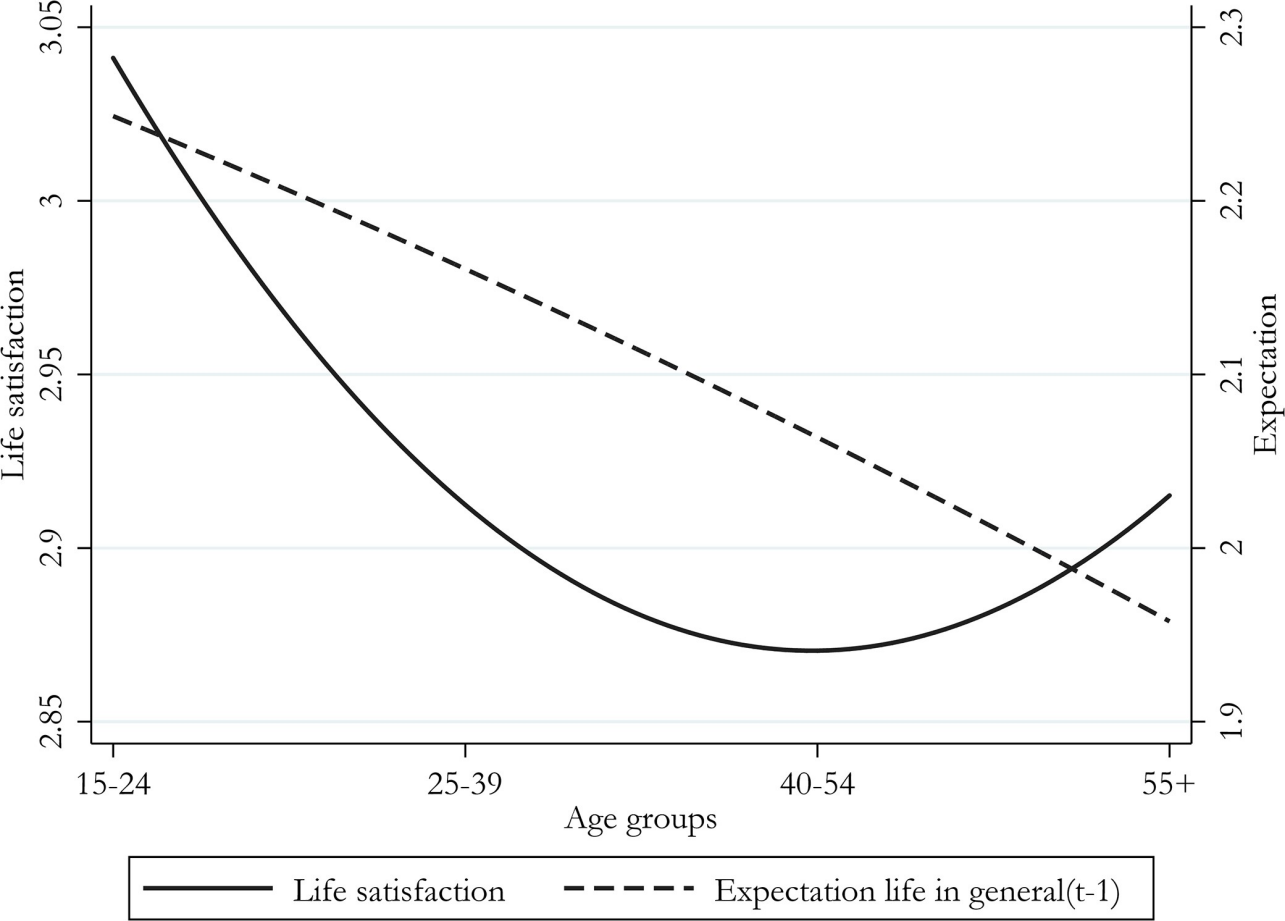
Aggregate life satisfaction and expectations, by age group.

Fig 2 shows the trend of life satisfaction and expectations about life as a whole for four different age groups. The graph shows that expectations are on average higher for younger age groups, and decrease progressively for older age groups. The expectation line is above 2 (indicating that individuals do on average expect a better life in the future) until about the age group 40–54, and it then goes below 2 thereafter (indicating expectations of a worse future). This evidence is in line with the model’s main assumption that the aspiration variable (here proxied by expectations about the future) decreases over time, shifting from optimism bias to pessimism bias throughout the life cycle (see Eq 3, in section 3). This pattern is consistent with the main idea and recent empirical evidence on unmet aspiration theory [19, 21, 59].

**Fig 3:**
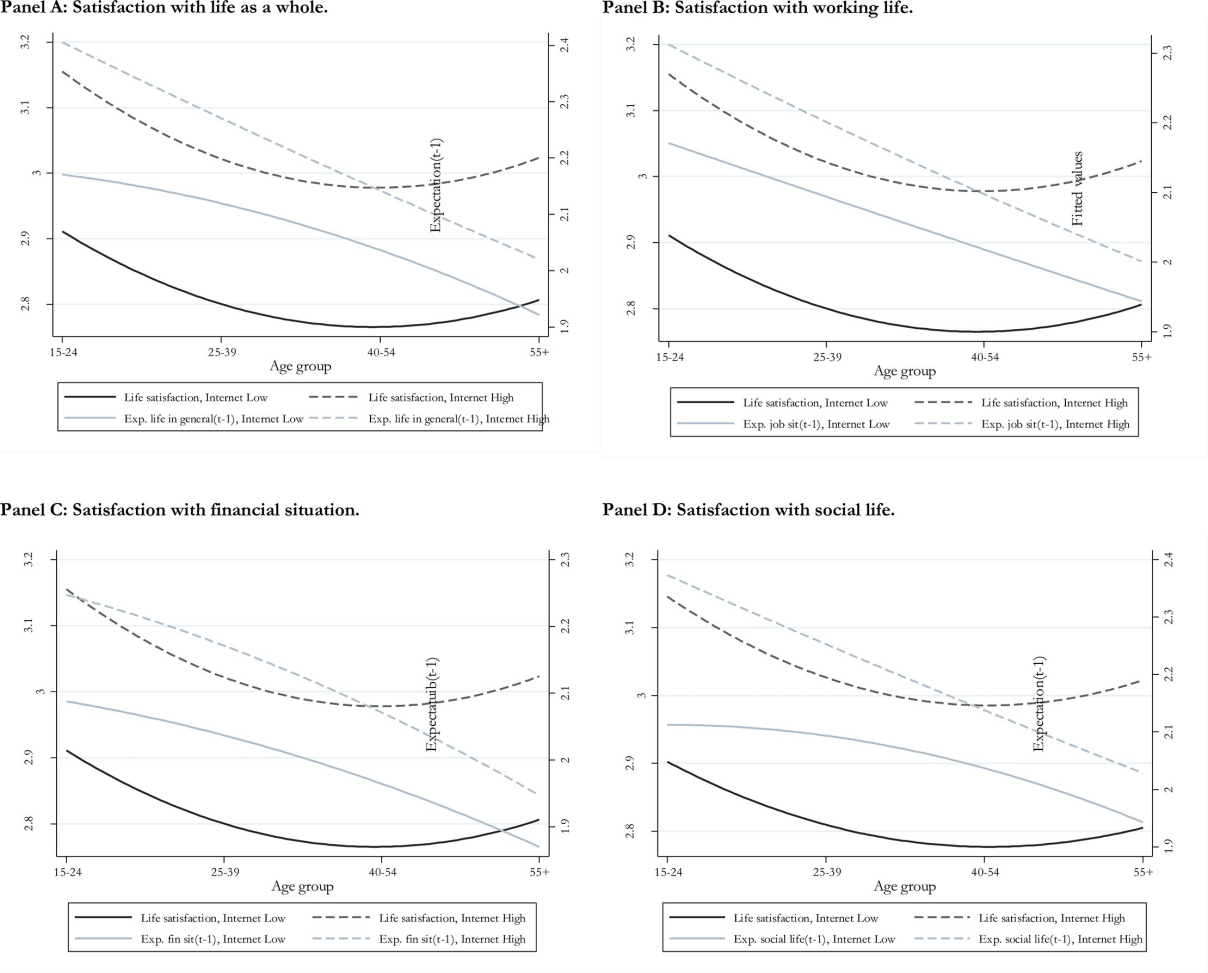
Aggregate life satisfaction and expectations, by age group and Internet users group. Panel A: Satisfaction with life as a whole. Panel B: Satisfaction with working life. Panel C: Satisfaction with financial situation. Panel D: Satisfaction with social life.

Fig 3 depicts the trends of expectations for different age groups, and comparing active Internet users to less active (or sporadic) users. The four panels in Fig 3 focus on expectations about life as a whole, working life, financial situation and social life, respectively. These figures consistently illustrate two patterns. The first is that, for all age groups, Internet users have on average higher expectations than non-users. This is in line with recent literature suggesting that Internet increases aspirations [12, 13, 60]. The second pattern is that in all four figures the expectation variable decreases with age, and this decline is relatively steeper for active Internet users than for sporadic users. This is consistent with the main property of our model that Internet use affects aspirations more strongly for individuals in younger and in older age groups (see Eq 8, in section 3).

Further, the figures also show that the point at which the life satisfaction curve meets the expectation line comes earlier for Internet users than for non-users. This point indicates the stage at which expectations begin to be lower than realized life satisfaction, and hence when optimism bias of younger ages is substituted by pessimism bias typical of older life stages. On the whole, this evidence is consistent with the main properties of the model presented in section 3, suggesting that the effects of Internet on the U-shape of life can be explained in terms of aspiration effects and their evolution over the life cycle. Further, comparing the four panels in Fig 3, it is interesting to observe the remarkable similarities between the patterns for life satisfaction as a whole, working life, financial conditions and social life. The latter domain (Fig 3, panel D) is the one for which Internet use seems to have stronger effects on aspiration levels. However, the overall pattern emerging from these four diagrams is basically the same.

To corroborate this interpretation, it would be interesting to analyze more specifically the type of activities that individuals carry out on the Internet, and the extent to which these are related to their aspirations and subjective well-being. In our econometric analysis, we are not able to investigate this point because information about different types of Internet activities is not available for the Eurobarometer surveys that we have used to construct our dataset. However, we can provide some descriptive evidence based on only some of the Eurobarometer surveys, which asked individuals the extent to which they use Internet to communicate through social networks, and to watch TV online. Previous research suggests that these two online activities can affect individuals’ aspirations. A11 Table shows the share of respondents that use social networks and TV streaming, and the expectations that these respondents report about their life satisfaction in the future. In line with our theoretical framework, the table indicates that active users of online social networks and of TV streaming are much more likely to have higher aspirations about their life satisfaction in the future than to expect a worsening of their life satisfaction.

### Other explanations

Could our empirical result of a moderation effect of Internet on life satisfaction be explained by a different mechanism that is not related to unmet aspirations? According to the model presented in section 3, this is theoretically possible. As noted above in relation to Eq 6, it would in fact be possible to assume that life satisfaction related to one given domain of life changes over the life cycle following a non-monotonic relationship, thus generating a U-shaped relationship between age and well-being (even in the absence of forecast errors and unmet aspirations).

Let us consider three main domains of life and three possible alternative explanations of our empirical results. First, let us consider income conditions and the consumption domain. Can consumption patterns explain the U-shape of life? According to extant research, the answer is no. Empirical research consistently shows that lifetime consumption patterns follow an *inverted* U-shape, with a maximum around midlife [61]. Internet may facilitate financial transactions and foster online consumption, but it is not reasonable to think that the utility derived from digital consumption may be such to generate a U-shape of life for active Internet users (other things being equal). Hence, we think that this first explanation is not plausible.

Second, shifting the focus to the social life domain, it would be possible to think that individuals value this differently throughout the life cycle. Social life could be regarded as an important dimension of life satisfaction at younger ages, less important during midlife (when many individuals shift their focus to working life and career objectives), and then becoming again more important at later stages of life. If the use of Internet facilitates social life activities (e.g. through social media and online communication platforms), this mechanism would be consistent with the empirical pattern that we have pointed out in the previous section, namely a steeper U-shape of life for active Internet users.

Third, focusing on working life, one could assume that this domain is a source of stress and responsibilities for individuals, and that these unpleasant effects are relatively stronger during midlife than at earlier and later phases of life. Further, an intensive working life does by definition reduce leisure time devoted to social life. This argument would also generate a U-shape between age and well-being. The empirical evidence available up to date indicates that Internet use for professional purposes leads to time-saving effects at work, and that it may also increase employees’ autonomy and flexibility [8]. Hence, this argument would also be consistent with our main empirical result, since the use of Internet at work may potentially improve life satisfaction and free time to be devoted to more rewarding leisure and social activities.

We think that the two possible explanations noted here (focusing on social life and working life respectively) are both consistent with the empirical result pointed out in this paper, and that they may arguably represent additional mechanisms that can explain moderation effects of Internet on the U-shape of life–in addition to the main explanation that we have focused on in this paper based on unmet aspirations theory. These possible mechanisms explaining the effects of Internet on the U-shape of life should be analyzed further and empirically tested in future research.

## 7. Conclusions

Empirical research has consistently shown that the relationship between age and life satisfaction is U-shaped. The present study has investigated whether, and the extent to which, Internet use moderates the U-shape of life. According to recent research [19, 22], one possible explanation of the U-shape relationship is related to unmet aspirations, pointing out that individuals make systematic forecast errors when they form expectations about future life satisfaction, and that these errors indicate optimism bias at early stages of life, and pessimism bias at older ages. Since unmet aspirations depress life satisfaction for younger individuals, and, by contrast, unexpected well-being fosters life satisfaction for older people, this theory can explain the U-shape pattern that has been observed and confirmed in previous empirical studies.

The present paper takes this theory as a conceptual framework, and it extends it by investigating the effects of Internet use. Our main proposition is that Internet tends to increase aspirations, and that this effect will be stronger for more vulnerable age groups, such as younger individuals and older adults. Internet use would thus make the U-shape relationship steeper, i.e. exacerbating optimism bias for the younger and pessimism bias for the older.

To empirically investigate this proposition, we used the Eurobarometer surveys for the years 2010 to 2016, and exploited exogenous variation in broadband Internet take-up across European countries and regions to identify the causal effects of Internet use on well-being for different age groups. The results of 2SLS bivariate ordered probit estimations are twofold. First, Internet use has a positive and significant effect on subjective well-being. Second, Internet use does also moderate the U-shaped relationship between age and well-being by making it steeper. Specifically, we find that Internet users experience a more pronounced decrease in reported life satisfaction in their younger adult life, and then an earlier and stronger recovery after the turning point (midlife crisis).

According to our model, the interpretation of this result is related to the effects of Internet on individuals’ aspirations, and how these effects differ for distinct age groups. As noted above, our dataset does not enable to carry out a proper empirical test of unmet aspirations theory at the individual level. However, we have reported aggregate (country-level) evidence showing that our empirical results are consistent with the main predictions of the unmet aspirations model. This evidence shows in fact that: (1) aspirations decline over the life cycle; (2) active Internet users have on average higher aspirations than less active users; (3) the effect of Internet use on aspirations is stronger for younger and older groups. In short, this aggregate evidence corroborates an unmet aspirations explanation of our econometric results.

However, as discussed above, it is also important to acknowledge that the U-shape of life could in principle be explained by other mechanisms, such as e.g. changing preferences and values over the life cycle. Therefore, we cannot rule out the possibility that our econometric results on the moderation effects of Internet may be explained not only by aspiration-related patterns, but also by age-specific effects of Internet in specific domains of life. This calls for further research investigating how the effects of Internet on well-being vary with age, employing individual-level data and variables that enable a test of different possible explanations.

Although the focus of this paper has been on age-specific patterns of Internet use and well-being, our findings may in principle also have relevance for the study of income-specific patterns, which affect the relationship between aspirations and well-being [62]. In particular, our study may be related to the emerging literature on aspirations and poverty. Dalton et al. [63] shows that poverty strengthens the effects of the behavioral bias that leads to aspirations failure. It is reasonable to think that poor people, which have now increasing access to internet, may for this reason raise their aspirations, and therefore increase their aspiration failure even further. The study of Internet use may thus be a relevant dimension to extend the literature on aspirations and poverty in future research.

By empirically showing that the effects of Internet are remarkably heterogeneous among individuals of different ages, the present work has also some important implications for policy. Digitalization is currently a recurrent theme of societal debate, and an important objective for policy. Many countries are actively investing large amounts of public resources to foster digitalization through Internet access and infrastructures (see e.g. the EU Digital Agenda program that has been considered in this paper). However, research-based evidence on how these policies affect different socio-demographic groups of the population is still limited. Our empirical findings indicate that the effects of increased access to Internet may be particularly positive for the well-being of older adults, and much less so for younger age individuals. For the latter, it is therefore important that digital infrastructures policies are combined with the development of appropriate regulations, ethical standards and education policies that may mitigate the risks for younger Internet users.

## Supporting information

S1 File Online appendix: Results for region-level instrument. (DOCX)
