# Supplementary material for: Internet, unmet aspirations and the U-shape of life
Source: PLoS One. 2020 Jun 5;15(6):e0233099. doi: 10.1371/journal.pone.0233099 (PMC7274442; doi:10.1371/journal.pone.0233099)
Supplement: S1 File — (DOCX) [file pone.0233099.s001.docx]

**Internet, unmet aspirations and the U-shape of life**

**Fulvio Castellacci^*^ and Henrik Schwabe**

University of Oslo,

Postboks 1108, 0317 Oslo, Norway

^*^Corresponding author: fulvio.castellacci@tik.uio.no

Manuscript revised and resubmitted to the *PLOS ONE*, April 2020

**Supporting information.**

**Online appendix: Results for region-level instrument**

**Table A.1. Descriptive statistics.**

|  |  | **Mean** | **St.dev.** | **Min** | **Max** | **Obs** |  |
| --- | --- | --- | --- | --- | --- | --- | --- |
|  |  |  |  |  |  |  |  |
|  | Life satisfaction | 2.92 | 0.81 | 1 | 4 | 139,865 |  |
|  | Internet use | 5.46 | 2.25 | 1 | 7 | 139,865 |  |
|  | Age | 47.18 | 18.15 | 15 | 99 | 139,865 |  |
|  | Women | 0.52 | 0.50 | 0 | 1 | 139,865 |  |
|  | House/apartment ownership | 0.74 | 0.44 | 0 | 1 | 139,865 |  |
|  | Up to 15 years of education | 0.25 | 0.43 | 0 | 1 | 139,865 |  |
|  | 16-19 years of education | 0.43 | 0.50 | 0 | 1 | 139,865 |  |
|  | 20+ years of education | 0.32 | 0.47 | 0 | 1 | 139,865 |  |
|  | Unemployment | 0.10 | 0.30 | 0 | 1 | 139,865 |  |
|  | Divorced | 0.07 | 0.26 | 0 | 1 | 139,865 |  |
|  | Widow | 0.08 | 0.28 | 0 | 1 | 139,865 |  |
|  | Living in a rural area or village | 0.33 | 0.47 | 0 | 1 | 139,865 |  |
|  | Living in a small/middle sized town | 0.39 | 0.49 | 0 | 1 | 139,865 |  |
|  | Living in a large town | 0.28 | 0.45 | 0 | 1 | 139,865 |  |
|  | White-collar | 0.36 | 0.48 | 0 | 1 | 139,865 |  |
|  | Blue-collar | 0.14 | 0.35 | 0 | 1 | 139,865 |  |
|  | HH broadband connection | 0.72 | 0.13 | 0.20 | 0.95 | 139,865 |  |
|  |  |  |  |  |  |  |  |

**Table A.2. Timing of broadband Internet variation.**

|  | **2012** | **2013** | **2014** | **2015** | **2016** |
| --- | --- | --- | --- | --- | --- |
| Education level | 0.017 | -0.028 | -0.006 | -0.051* | 0.015 |
| Women | 0.014 | 0.088 | 0.032 | -0.005 | -0.009 |
| Urbanization | 0.002 | 0.022 | -0.008 | 0.011 | 0.012 |
| Unemployment | -0.254 | 0.105 | 0.045 | -0.003 | 0.131 |
| Age | -0.000 | 0.004* | 0.000 | 0.003 | 0.002 |
| House/apartment ownership | 0.004 | -0.051 | -0.074 | -0.016 | -0.062* |
| Divorces | -0.149 | -0.356** | -0.440** | -0.312** | -0.155 |
| Widowers | -0.060 | 0.069 | 0.093 | -0.222 | 0.172 |
| Blue-collar jobs | -0.027 | 0.096 | -0.004 | 0.036 | -0.030 |
| White-collar jobs | -0.229 | 0.240** | 0.100 | -0.062 | 0.037 |

*Note:* The table reports estimated coefficients from regressions of the annual growth rate of broadband Internet for the years 2012 to 2016 on the level of covariates. The regression is based on a panel of 598 region-year observations.

* p<0.10; ** p<0.05; *** p<0.01

**Table A.3. Balancing regressions.**

|  |  | **Financial situation** | **Education** | **Unemployment** |  |
| --- | --- | --- | --- | --- | --- |
|  | HH broadband connection t-1 | -0.014 | -0.099 | -0.036 |  |
|  |  | (0.072) | (0.107) | (0.037) |  |
|  | HH broadband connection t+1 | 0.113 | 0.064 | -0.173*** |  |
|  |  | (0.105) | (0.110) | (0.049) |  |

The table reports the estimated coefficients of lagged and lead broadband Internet take-up in three balancing regressions. These regress the dependent variables financial situation, education and unemployment, respectively, on the set of covariates included in the model plus the lead and lagged instruments. Heteroskedasticity-robust standard errors in parentheses. All regressions include a full set of control variables, time dummies and country fixed effects. * p<0.10; ** p<0.05; *** p<0.01.

**Table A.4. First stage results. Baseline estimations and balancing tests.**

|  |  | **Baseline** | **Balancing test** |  |
| --- | --- | --- | --- | --- |
|  | HH broadband connection t-1 | 0.789*** | 0.792*** |  |
|  |  | (0.217) | (0.214) |  |
|  | Age | -0.045*** | -0.045*** |  |
|  |  | (0.008) | (0.008) |  |
|  | Age squared | -0.000*** | -0.000*** |  |
|  |  | (0.000) | (0.000) |  |
|  | Women | -0.096*** | -0.095*** |  |
|  |  | (0.020) | (0.020) |  |
|  | House/apartment ownership | 0.209*** | 0.211*** |  |
|  |  | (0.022) | (0.022) |  |
|  | 16-19 years of education | 0.638*** | 0.639*** |  |
|  |  | (0.070) | (0.070) |  |
|  | 20+ years of education | 1.177*** | 1.178*** |  |
|  |  | (0.069) | (0.069) |  |
|  | Unemployment | -0.308*** | -0.308*** |  |
|  |  | (0.039) | (0.039) |  |
|  | White-collar job | 0.501*** | 0.499*** |  |
|  |  | (0.068) | (0.068) |  |
|  | Blue-collar job | -0.083* | -0.082 |  |
|  |  | (0.049) | (0.049) |  |
|  | Divorced | -0.000 | -0.000 |  |
|  |  | (0.036) | (0.036) |  |
|  | Widowed | -0.558*** | -0.558*** |  |
|  |  | (0.047) | (0.047) |  |
|  | Living in a rural area or village | -0.182*** | -0.183*** |  |
|  |  | (0.023) | (0.023) |  |
|  | Living in a large town | 0.165*** | 0.166*** |  |
|  |  | (0.021) | (0.021) |  |
|  | _cons | 6.518*** | 6.519*** |  |
|  |  | (0.214) | (0.218) |  |
|  |  |  |  |  |
|  | N | 139,865 | 139,865 |  |
|  | F-value (instrument) | 13.22 |  |  |

Robust standard errors clustered at the NUTS1 level in parentheses. The second column include a full set of controls that was averaged and lagged over region-years (i.e. $\bar{{x'}_{r,t}}$, and $\bar{{x'}_{r,t-1}}$) time dummies and region fixed effects. In additional regressions not reported here, we have also added country-level growth rates of all covariates. In additional regressions not reported here, we have also added country-level growth rates of all covariates. In additional regressions not reported here, we have also added country-level growth rates of all covariates. * p<0.10; ** p<0.05; *** p<0.01.

**Table A.5. LATE results: compliers for different age groups.**

|  |  | **P[X=x]** | **Coefficient of HH broadband connection** |  |
| --- | --- | --- | --- | --- |
| **Age groups** |  |  |  |  |
|  | Young (15-24) | 0.092 | 1.205** |  |
|  | Younger adults (25-39) | 0.226 | 1.900*** |  |
|  | Middle-aged (40-54) | 0.259 | 1.721*** |  |
|  | Older adults (55+) | 0.423 | 0.194 |  |

*Note:* Column 1 reports the relative shares of each age group of the total sample. The second column reports the first stage coefficients on our instrument. The regressions include time and region dummies. * p<0.10; ** p<0.05; *** p<0.01.

**Table A.6. Second stage results. Baseline estimations.**

|  | **Baseline** | **Pre-reform trend** | **Placebo** | **Cohort** | **Full model** |
| --- | --- | --- | --- | --- | --- |
| Internet use | 0.059*** | 0.059*** |  | 0.072*** | 0.120*** |
|  | (0.006) | (0.006) |  | (0.007) | (0.029) |
| Internet use X age |  |  |  |  | -0.004 |
|  |  |  |  |  | (0.003) |
| Internet use X age squared |  |  |  |  | 0.000** |
|  |  |  |  |  | (0.000) |
| Age | -0.043*** | -0.043*** | -0.045*** | -0.042*** | -0.047*** |
|  | (0.002) | (0.002) | (0.002) | (0.003) | (0.012) |
| Age squared | 0.000*** | 0.000*** | 0.000*** | 0.000*** | 0.000** |
|  | (0.000) | (0.000) | (0.000) | (0.000) | (0.000) |
| Women | 0.029*** | 0.029*** | 0.024*** | 0.033*** | 0.049*** |
|  | (0.007) | (0.007) | (0.008) | (0.007) | (0.009) |
| House/apartment ownership | 0.198*** | 0.198*** | 0.208*** | 0.189*** | 0.163*** |
|  | (0.017) | (0.017) | (0.017) | (0.017) | (0.025) |
| 16-19 years of education | 0.022 | 0.022 | 0.060*** | 0.024 | -0.075 |
|  | (0.016) | (0.016) | (0.019) | (0.016) | (0.048) |
| 20+ years of education | 0.179*** | 0.179*** | 0.249*** | 0.182*** | 0.016 |
|  | (0.019) | (0.019) | (0.022) | (0.020) | (0.087) |
| Unemployment | -0.518*** | -0.518*** | -0.532*** | -0.486*** | -0.445*** |
|  | (0.021) | (0.021) | (0.022) | (0.020) | (0.030) |
| White-collar job | 0.077*** | 0.078*** | 0.108*** | 0.109*** | 0.103** |
|  | (0.015) | (0.015) | (0.015) | (0.016) | (0.052) |
| Blue-collar job | -0.091*** | -0.091*** | -0.097*** | -0.054*** | -0.034 |
|  | (0.017) | (0.017) | (0.017) | (0.018) | (0.021) |
| Divorced | -0.285*** | -0.285*** | -0.285*** | -0.287*** | -0.291*** |
|  | (0.015) | (0.015) | (0.015) | (0.015) | (0.016) |
| Widowed | -0.251*** | -0.251*** | -0.281*** | -0.238*** | -0.101** |
|  | (0.015) | (0.015) | (0.015) | (0.015) | (0.040) |
| Living in a rural area or village | 0.017 | 0.018 | 0.006 | 0.014 | 0.036** |
|  | (0.013) | (0.013) | (0.013) | (0.013) | (0.018) |
| Living in a large town | -0.002 | -0.002 | 0.007 | -0.009 | -0.021 |
|  | (0.016) | (0.016) | (0.017) | (0.016) | (0.018) |
|  |  |  |  |  |  |
| Pre-reform trend in life satisfaction (linear) |  | -0.022 |  |  |  |
|  |  | (0.064) |  |  |  |
| HH broadband connection t-1 |  |  | 0.013 |  |  |
|  |  |  | (0.261) |  |  |
| HH broadband connection t+1 |  |  | 0.672 |  |  |
|  |  |  | (0.419) |  |  |
|  |  |  |  |  |  |
| N | 139,865 | 139,865 | 137,978 | 139,865 | 139,865 |
|  |  |  |  |  |  |
| Atanhrho | 0.016 |  |  | -0.007 |  |
|  | (0.015) |  |  | (0.015) |  |

Robust standard errors clustered at the NUTS1 level in parentheses. All regressions include time dummies and region fixed effects. * p<0.10; ** p<0.05; *** p<0.01.

**Table A.7. Second stage results. Separate estimations for different age groups.**

|  | ***15-24*** | ***25-39*** | ***40-54*** | ***55+*** |
| --- | --- | --- | --- | --- |
| Internet use | 0.087*** | 0.033*** | 0.016 | 0.038*** |
|  | (0.023) | (0.013) | (0.010) | (0.009) |
| Women | -0.023 | 0.065*** | 0.058*** | 0.012 |
|  | (0.024) | (0.015) | (0.014) | (0.012) |
| House/apartment ownership | 0.185*** | 0.164*** | 0.208*** | 0.211*** |
|  | (0.030) | (0.023) | (0.026) | (0.021) |
| 16-19 years of education | -0.048 | 0.029 | 0.077** | 0.098*** |
|  | (0.050) | (0.038) | (0.030) | (0.021) |
| 20+ years of education | -0.049 | 0.218*** | 0.260*** | 0.276*** |
|  | (0.066) | (0.041) | (0.042) | (0.031) |
| Unemployment | -0.606*** | -0.461*** | -0.377*** | -0.511*** |
|  | (0.064) | (0.038) | (0.030) | (0.030) |
| White-collar job | -0.114** | 0.139*** | 0.322*** | 0.035 |
|  | (0.056) | (0.032) | (0.034) | (0.027) |
| Blue-collar job | -0.183*** | -0.054 | 0.120*** | -0.138*** |
|  | (0.067) | (0.035) | (0.029) | (0.021) |
| Divorced | -0.235* | -0.371*** | -0.292*** | -0.286*** |
|  | (0.136) | (0.034) | (0.023) | (0.019) |
| Widowed | 0.154 | -0.353*** | -0.321*** | -0.151*** |
|  | (0.212) | (0.102) | (0.040) | (0.016) |
| Living in a rural area or village | 0.077*** | 0.033 | 0.001 | -0.008 |
|  | (0.029) | (0.021) | (0.016) | (0.016) |
| Living in a large town | 0.009 | 0.022 | -0.032 | -0.012 |
|  | (0.030) | (0.022) | (0.028) | (0.019) |
|  |  |  |  |  |
| N | 12863 | 31645 | 36210 | 59147 |

Robust errors clustered at the NUTS1 level in parentheses. All regressions include time dummies and region fixed effects. * p<0.10; ** p<0.05; *** p<0.01.

**Table A.8. Second stage results. Separate estimations for different Internet use groups.**

|  | ***No access*** | ***Never use*** | ***Less often*** | ***2-3 times a month*** | ***About once a week*** | ***2-3 times a week*** | ***Every day*** |
| --- | --- | --- | --- | --- | --- | --- | --- |
| Age | -0.029*** | -0.024*** | -0.036*** | -0.045*** | -0.041*** | -0.047*** | -0.051*** |
|  | (0.007) | (0.005) | (0.012) | (0.017) | (0.010) | (0.005) | (0.003) |
| Age squared | 0.000*** | 0.000*** | 0.000*** | 0.000*** | 0.000*** | 0.000*** | 0.001*** |
|  | (0.000) | (0.000) | (0.000) | (0.000) | (0.000) | (0.000) | (0.000) |
| Women | 0.051 | 0.035** | 0.077* | 0.051 | 0.130*** | 0.017 | 0.030*** |
|  | (0.032) | (0.018) | (0.046) | (0.066) | (0.044) | (0.020) | (0.009) |
| Financial situation | 0.251*** | 0.173*** | 0.054 | 0.149* | 0.130** | 0.186*** | 0.210*** |
|  | (0.037) | (0.025) | (0.058) | (0.084) | (0.054) | (0.034) | (0.021) |
| 16-19 years of education | 0.175*** | 0.110*** | 0.105* | 0.102 | 0.023 | 0.073** | -0.068*** |
|  | (0.034) | (0.021) | (0.062) | (0.079) | (0.060) | (0.031) | (0.024) |
| 20+ years of education | 0.385*** | 0.275*** | 0.239** | 0.205** | 0.047 | 0.177*** | 0.104*** |
|  | (0.042) | (0.029) | (0.094) | (0.103) | (0.077) | (0.039) | (0.027) |
| Unemployment | -0.436*** | -0.411*** | -0.522*** | -0.431*** | -0.265*** | -0.559*** | -0.504*** |
|  | (0.043) | (0.034) | (0.077) | (0.123) | (0.084) | (0.055) | (0.028) |
| White-collar job | 0.146** | 0.176*** | 0.276*** | 0.087 | 0.175*** | 0.086** | 0.106*** |
|  | (0.063) | (0.041) | (0.071) | (0.101) | (0.063) | (0.039) | (0.023) |
| Blue-collar job | 0.026 | 0.004 | -0.026 | 0.060 | 0.024 | -0.111*** | -0.075*** |
|  | (0.055) | (0.029) | (0.067) | (0.109) | (0.079) | (0.034) | (0.027) |
| Divorced | -0.223*** | -0.282*** | -0.358*** | -0.183 | -0.342*** | -0.314*** | -0.299*** |
|  | (0.044) | (0.029) | (0.075) | (0.113) | (0.075) | (0.033) | (0.020) |
| Widowed | -0.140*** | -0.176*** | -0.208** | -0.166 | -0.255*** | -0.253*** | -0.337*** |
|  | (0.031) | (0.020) | (0.094) | (0.104) | (0.067) | (0.053) | (0.028) |
| Living in a rural area or village | 0.031 | 0.038 | 0.084 | 0.024 | -0.004 | 0.027 | 0.015 |
|  | (0.041) | (0.025) | (0.054) | (0.092) | (0.060) | (0.030) | (0.013) |
| Living in a large town | -0.025 | -0.050** | -0.136* | -0.042 | -0.126* | -0.017 | 0.012 |
|  | (0.055) | (0.024) | (0.081) | (0.104) | (0.072) | (0.034) | (0.017) |
|  |  |  |  |  |  |  |  |
|  |  |  |  |  |  |  |  |
| N | 9237 | 28738 | 2972 | 1697 | 3816 | 12418 | 80987 |
|  |  |  |  |  |  |  |  |

Heteroskedasticity-robust standard errors in parentheses. All regressions include time dummies and country fixed effects. * p<0.10; ** p<0.05; *** p<0.01.

**Table A.9. Moderation effects of Internet use on the location of turning point of U-shape.**

| **Internet use intensity** | **Turning point of U-shape** |
| --- | --- |
| No Internet access | 50.76 |
| Never use Internet | 47.83 |
| Less than 2-3 times per month | 45.05 |
| 2-3 times per month | 42.41 |
| About once a week | 39.91 |
| 2-3 times per week | 37.52 |
| Everyday | 35.25 |

**Table A.10. Moderation effects of Internet use on the U-shape curvature.**

|  | **Internet use category** | | |  |  |  |  |
| --- | --- | --- | --- | --- | --- | --- | --- |
|  | ***1*** | ***2*** | ***3*** | ***4*** | ***5*** | ***6*** | ***7*** |
| **Age** |  |  |  |  |  |  |  |
| ***25*** | -0.014 | -0.012 | -0.016 | -0.021 | -0.018 | -0.023 | -0.025 |
| ***35*** | -0.009 | -0.007 | -0.009 | -0.011 | -0.008 | -0.013 | -0.014 |
| ***45*** | -0.003 | -0.002 | -0.001 | -0.001 | 0.001 | -0.003 | -0.004 |
| ***55*** | 0.003 | 0.003 | 0.007 | 0.009 | 0.010 | 0.007 | 0.007 |
| ***65*** | 0.009 | 0.008 | 0.015 | 0.019 | 0.020 | 0.017 | 0.018 |
| ***85*** | 0.020 | 0.018 | 0.030 | 0.038 | 0.039 | 0.036 | 0.039 |

**A11 Table. Internet use activities (social networks; TV streaming) and expectations about future life satisfaction.**

|  | **Social networks** | | | **TV streaming** | | |
| --- | --- | --- | --- | --- | --- | --- |
| Expectation: Life in general | *Worse* | *Same* | *Better* | *Worse* | *Same* | *Better* |
| Non-users | 58 % | 48 % | 25 % | 74 % | 67 % | 50 % |
| Active users | 42 % | 52 % | 75 % | 26 % | 33 % | 50 % |
